# Supplementary material for: Compromised Biomechanical Properties, Cell–Cell Adhesion and Nanotubes Communication in Cardiac Fibroblasts Carrying the Lamin A/C D192G Mutation
Source: Int J Mol Sci. 2021 Aug 25;22(17):9193. doi: 10.3390/ijms22179193 (PMC8431729; doi:10.3390/ijms22179193)

Supplementary Table S1: Number of measurements and analysed samples during AFM experiments.

	Fibro-CT	Fibro-WT	Fibro-MT	Fibro-WT+Gap27
cell number	51	87	67	56
curves number	178	261	201	168
analyzed curves	152	221	177	122
flat curves	2	0	14	27
rupture events	230	1183	540	321
nanotubes events	199	783	415	192
average rupture events/curve	4.51	5.35	3.05	2.63
average nanotubes events/curve	3.90	3.54	2.34	1.57

Supplementary Figure S1: NRVFs dimensions. A) Cell length. B) Cell width. C) Comparison of TNT width, (* *P* < 0.0001, ** *P* < 0.001).**

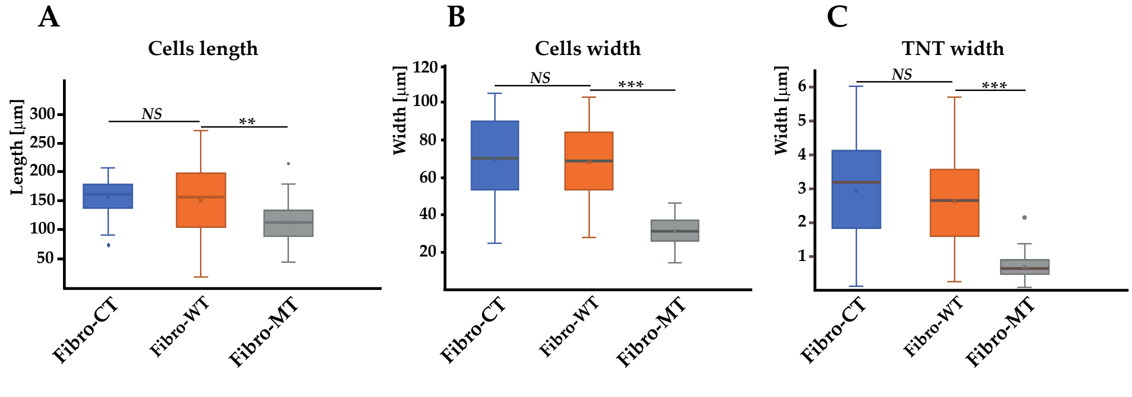

Supplement: Supplementary file 1 [file ijms-22-09193-s001.zip › Supplementary Table 1_def.pdf]
